# Upregulation of Long Noncoding RNA SPRY4-IT1 Modulates Proliferation, Migration, Apoptosis, and Network Formation in Trophoblast Cells HTR-8SV/neo

**DOI:** 10.1371/journal.pone.0079598

**Published:** 2013-11-06

**Authors:** Yanfen Zou, Ziyan Jiang, Xiang Yu, Ming Sun, Yuanyuan Zhang, Qing Zuo, Jing Zhou, Nana Yang, Ping Han, Zhiping Ge, Wei De, Lizhou Sun

**Affiliations:** 1 Department of Obstetrics and Gynecology, the First Affiliated Hospital of Nanjing Medical University, Nanjing, JiangSu Province, China; 2 Nanjing Medical University, Nanjing, JiangSu Province, China; Brigham and Women's Hospital, United States of America

## Abstract

SPRY4-IT1 has been reported to have extremely high expression in normal placenta tissues. It is a Long noncoding RNA (lncRNA), which is associated with cell growth, migration, invasion, and apoptosis in melanoma. A 2.8-fold increase of SPRY4-IT1 expression was validated by Real-time reverse transcription-polymerase chain reaction (qRT-PCR) in severe preeclamptic placenta as compared with that of the normal ones (n=25) in this study. Furthermore, the role of SPRY4-IT1 in proliferation, migration, apoptosis, and network formation ability of trophoblast cells HTR-8/SVneo was assessed. Suppression of SPRY4-IT1 using siRNA treatment and its overexpression using plasmid targeting SPRY4-IT1 were performed in order to explore the biological function of SPRY4-IT1 in the development and progression of trophoblast cells HTR-8/SVneo, in vitro. The results showed that SPRY4-IT1 knockdown enhanced the cell migration and proliferation, and reduced the response of cells to apoptosis. However, exogenous SPRY4-IT1 overexpression significantly decreased the cell migration and proliferation, while increased cell apoptosis. Our study showed for the first time that aberrant expression of lncRNA SPRY4-IT1 might contribute to the abnormal condition of trophoblast cells HTR-8/SVneo. Therefore, we proposed SPRY4-IT1 as a novel lncRNA molecule, which might be associated with the pathogenesis of preeclampsia and might provide a new target for its early diagnosis and treatment.

## Introduction

Preeclampsia, as one of the most common pathologic complications of pregnancy, afflicts approximately 3-5% [1] of the pregnancies and is a leading contributor to perinatal morbidity and pregnancy-associated-mortality, especially in developing countries. The criteria for preeclampsia diagnosis are: a new onset of hypertension (blood pressure≥140/90 mmHg in previously normotensive women) and proteinuria (≥300 mg/24-hour urine collection or random urine protein (+)) after the 20th week of the pregnancy. The delivery of the baby and placenta is the only curative treatment to reverse the syndrome [2]. Because of the adverse outcome of this disease, continuously growing number of studies has been focusing on this area; however, the specific pathogenesis of this disorder remains to be elucidated. 

There have been many theories associated with the pathogenesis of preeclampsia, such as inflammatory cytokines [3–5], endothelial dysfunction [6], imbalance between proangiogenic and antiangiogenic factors [7,8], oxidative stress [9,10], and finally genetic and dietary factors [11]. Among those, oxidative stress activates a number of signaling pathways that restore homeostasis but in case of failure, the apoptotic machinery might be activated [12]. Many studies have found that there was an increased level of villous trophoblast apoptosis in pregnancies complicated by preeclampsia [13]. Furthermore, in our previous studies, we revealed specific proteins such as CHOP in the endoplasmic reticulum stress pathway of apoptosis involved in the endoplasmic reticulum associated death (ERAD) in preeclampsia (article being contributed). It has been generally accepted that inadequate migration and invasion of placental trophoblast cells into the maternal spiral arteries could play a role in hypoxia of the placenta and impaired placentation [14]. Many efforts have been focused to elucidate the mechanisms of preeclampsia for many years; however, the detailed mechanisms associated with the process of trophoblast cell apoptosis, migration, and invasion in preeclampsia remain unclear.

During the process of maternal spiral artery remodeling in normal pregnancy, extra villous trophoblast (EVT) cells attach to and invade the maternal deciduas, where they undergo endovascular transformation. The normal muscular elastic structure of the maternal spiral arteries are replaced by fibrinoid material containing trophoblast, which results in conversion of the arteries into the large diameter, low resistance vessels that could provide steady perfusion of the villous trophoblast with maternal blood [15]. Failure of trophoblast invasion might cause insufficient remodeling of spiral arteries, which could play an important role in the pathogenesis of preeclampsia [16]. Remodeling of maternal spiral arteries involves trophoblast invasion and EVT differentiation [17].Previous reports have shown that primary cytotrophoblast and HTR-8/Svneo cells showed endothelial cell-like behavior in their ability to form tube-like networks when grown on matrigel [18]. Accordingly, we used an in vitro culture system to investigate the effects of SPRY4-IT1 on the HTR-8/Svneo cell network formation in order to reflect the function of SPRY4-IT1 on in vivo remodeling of maternal spiral arteries during preeclampsia.

Long noncoding RNAs (lncRNAs), as emerging molecules discovered in the last five years, have been reported to play a critical role in regulation of diverse cellular processes such as stem cell pluripotency, development, and cell growth. Also many studies have proposed that lncRNAs are closely associated with cell apoptosis and migration [19], such as HOTAIR [20–22], MEG3 [23], and MALAT1 [24] and might contribute to the behavior of trophoblast cells in preeclampsia. In 2011, Khaitan et al. reported that the long noncoding RNA SPRY4-IT1 could modulate the cells apoptosis, migration, and invasion in melanoma [19]. They identified and characterized lncRNAs that were differentially expressed in melanoma. knocking-down their expression resulted in defects in cell growth, invasion, and elevated the rates of apoptosis in melanoma cells [19]. Meanwhile, they also detected the expression level of lncRNA SPRY4-IT1 in many normal human tissues and revealed a high expression level in human placenta tissues. However, the underlying mechanism of lncRNA SPRY4-IT1 in regulating the behavior of trophoblast cells in preeclampsia is hardly clarified. 

In this study, we found a 2.8-fold increase in the SPRY4-IT1 expression in severe preeclampsia placental tissue as compared to that of the controls by qPCR. Therefore, we attempted to further investigate the effect of SPRY4-IT1 on HTR-8/SVneo cell proliferation, migration, and apoptosis as well as their network formation ability in preeclampsia. 

## Materials and Methods

### Patients and clinical sample collection

Placental tissues were obtained from primipara women aged 20–36 years, who underwent cesarean deliveries between 2011 and 2012 in the Department of Obstetrics and Gynecology of the People’s Hospital of Jiangsu Province, China. All tissues were immediately collected after the placenta delivery and were washed with sterile phosphate-buffered saline before being snap frozen in liquid nitrogen for future RNA and protein extraction. All of the experiments were approved by the Ethics Board of the First Affiliated Hospital of Nanjing Medical University. The informed consents were provided by all the patients and all the clinical investigations were conducted according to the principles expressed in the Declaration of Helsinki.

### Cell Culture and treatment

An immortalized first-trimester EVT cell line HTR-8/SVneo [25](kindly provided by Dr. Charles Graham, Queen’s University, Canada), which was derived from a short-lived primary EVT cell line was used in the present study. The cells could be characterized according to their expression of various morphological and functional markers (cytokeratin and human chorionic gonadotrophin) [26]. Resembling the behavior of trophoblast cells, many studies have been conducted based on the use of this cell line in order to simulate the behavior of trophoblast cells in pregnancy [27]. The HTR-8/SVneo cells were maintained in RPMI1640 medium, supplemented with 10% heat-inactivated fetal bovine serum (FBS), 100 U/ml penicillin and 100 ug/ml streptomycin in standard culture conditions (37°C in 5% humidified CO2 incubator). The cells were transiently transfected with si-RNAs after being sowed into the 6-well plates overnight. A scrambled negative control, a plasmid overexpressing SPRY4-IT1, and an empty vector, were cultured as well using the Lipofectamine 2000 transfection reagent (Invitrogen, Carlsbad, CA) and FuGENE^®^ HD Transfection Reagent (Roche, Germany) according to the manufacturer’s instructions, respectively. Forty eight hours after transfection, the cells were harvested to detect the overexpression or knockout efficiency via quantitative real-time PCR (qRT-PCR). For RNAi-mediated knockdown of SPRY4-IT1, three different Stealth siRNAs against SPRY4-IT1 were provided by Invitrogen. The target sequences for the si-SPRY4-IT1 included:si-SPRY4-IT1-1(CCCAGAATGTTGACAGCTGCCTCTT);si-SPRY4-IT1-2 (TGGAGGGTTATGGGAGCCTGTGAAT);and si-SPRY4-IT1-3(GCTTTCTGATTCCAAGGCCTATTAA) with the later having the highest inhibition efficiency. In order to ectopically express the SPRY4-IT1, the synthetic SPRY4-IT1 sequence (708 bp) was sub-cloned into the pEGFP-N1 plasmid vector. After the SPRY4-IT1 sequence was inserted into the vector, a sequencing analysis was conducted to make sure that this vector could specifically express SPRY4-IT1 (constructed by Invitrogen Inc.). 

### Cell proliferation assay

The proliferation capacity of the trophoblast cells was assessed by MTT assay (Sigma) according to the manufacturer’s instructions. The HTR-8/SVneo cells were plated into the 96-well plate after being transfected with si-RNAs targeting SPRY4-IT1 and plasmid overexpressing SPRY4-IT1. These cells were left in the culture for 24 h with five replicate wells at 10^4^ cells per well. They were then treated with 100 ug MTT by adding it to the medium after the cells were incubated for 6, 24, 48, 72, and 96 h. The incubation was then continued for another 4 h before the cell medium was removed, when dimethylsulfoxide (DMSO) was added for 10 minutes to lyse the cells. Finally, the absorbance was measured at 490 nm using an enzyme-linked immunosorbent assay plate reader. Each experiment was performed in triplicate.

### In Vitro Cell Migration Assays

The HTR-8/SVneo cells were resuspended in RPMI1640 medium containing 1% FBS, 24 hr after being transfected with the two groups as previously described. They were then added to the upper well of a transwell chamber (Millipore, Billerica, MA) with a membrane pore with an 8 um diameter at a density of 5×10^5^ cells/well. The medium containing 10% FBS was immediately placed into the lower well of the chamber as a chemo-attractant. After 24 h of incubation, the remaining cells were removed from the upper surface of the membrane by using a sterile cotton swab while those cells that had migrated to the lower surface were fixed and stained with 0.1% crystal violet. Finally, the number of migrated cells was examined using a digital microscopy. Cell numbers were calculated in five random fields for each chamber, and the average value was calculated. Each experiment was conducted in triplicate.

### In Vitro Network Formation Assay

Previous reports have shown that HTR-8/Svneo cells showed endothelial cell-like behavior in their ability to form tube-like networks when grown on matrigel [18]. We used an in vitro culture system to investigate the effect of SPRY4-IT1 on HTR-8/Svneo cell network formation. Transwell cell culture inserts (12 mm diameter, 0.4 mM pore size; Corning, Fisher Scientific UK Ltd., Loughborough, UK) were coated with 100 uL of the growth factor reduced Matrigel (BD Biosciences, Oxford, UK) and allowed to set at 37°C for at least 30 min. A total of 2.5×10^4^ transfected HTR-8/SVneo cells, resuspended in media containing 1% FBS, were plated into the upper layer of the chamber previously coated with matrigel. The cells were incubated at 37°C with 5% CO2 for 24 h. They were then washed three times in order to remove the scattered or the bridge-like cells that did not form networks. They were fixed in methanol, stained with crystal violet, and five areas from each well were image captured under a fluorescent microscope. The images were blinded and the number of cell–cell protracted contacts was counted as representations of the number of capillary-like networks present in each field. Each experiment was performed in duplicate. Data are presented as the average number of networks per field+SEM from at least three separate experiments

### RNA Extraction and Real-Time RT-PCR

Total RNA was extracted from approximately 0.1 g of placenta tissue or cells that were treated with Trizol reagent (Invitrogen Life Technologies). A Reverse Transcription Kit (Takara) was used for the synthesis of cDNA by adding 1 mg total RNA to the RT Reaction Mix. The amplification of cDNA was done by Power SYBR Green (Takara) in a total volume of 20 uL reaction mix for qPCR. According to the manufacturer’s instruction, the reverse transcription was performed at 37°C for 15 min, 85°C for 5 second. In order to normalize the results for the qPCR, the glyceraldehyde-3-phos-phate dehydrogenase (GAPDH) expressions were used. The sequence of the primers were as following: SPRY4-IT1 (Forward: 5’-AGCCACATAAATTCAGCAGA-3’, Reverse: 5’-CGATGTAGTAGGATTCCTTTCA-3’) and GAPDH (Forward: 5’-GACTCATGACCACAGTCCATGC-3’, Reverse: 5’-AGAGGCAGGGATGATGTTCTG-3’). An ABI 7500 was used to carry out the qPCR and data collections.

### Western Blotting analysis

The HTR-8/SVneo cells transfected with downregulating and overexpressing plasmid groups were lysed for protein extraction using mammalian reagent RIPA (Beyotime) supplemented with protease inhibitors cocktail (Roche) and phenylmethylsulfonyl-fluoride (Roche). The Bradford assay was used for the determination of protein concentration in each sample. Samples containing 50–100 micrograms of protein extractions were separated by 10% sodium dodecyl sulfate-polyacrylamide gel electrophoresis (SDS-PAGE). The separated proteins were transferred to 0.22 mm nitrocellulose (NC) or polyvinylidene difluoride membranes (Sigma) and were incubated with specific antibodies (Caspase-3, Cleaved Caspase-3, BCL-2, and BAX, all purchased from Cell Signaling Technology) at 1:1000 concentration. The secondary antibody was horseradish peroxidase-conjugated goat anti-rabbit IgG or goat anti-mouse IgG (1:1,000; Beijing ZhongShan Biotechnology CO., Beijing). Quantity One software (Bio-Rad) was used to quantify the intensity of autoradiogram protein bands and β-actin antibody (1:1000, Santa Cruz, CA, USA) was used as control. All experiments were repeated at least five times.

### Flow cytometry (FCM)

The HTR-8/SVneo cells were transiently transfected with downregulating (si-NC and si-SPRY4-IT1) and overexpressing (pEGFP-N1 and pEGFP-SPRY4-IT1) plasmid groups, respectively. After 48h, they were harvested using trypsin without EDTA, washed with PBS, resuspended in 1 ml binding buffer, and stained for 15 min with fluoresce inisothio-cyanate (FITC)-Annexin V and propidium iodide (PI) in the dark at room temperature, according to the manufacturer’s recommendations. The analysis of the cells was done by flow cytometry (FACScan; BD Biosciences) equipped with a CellQuest software (BD Biosciences). Cells were sorted into living, necrotic, early apoptotic, and late apoptotic cells. The relative ratio of early and late apoptotic cells were counted for further comparison. This assay was repeated more than three times.

### Terminal Deoxyribonucleotide Transferase-Mediated dUTP Nick-end Labeling (TUNEL)

The 3’-OH of the DNA fragments in apoptotic cells were labeled and stained by terminal dexynucleotidyl transferase (TdT)-mediated dUTP nick end labeling method using an apoptosis in situ detection kit (Roche, Germany), according to the manufacturer’s instructions. Fluorescent microscopy (Nikon Corporation, Tokyo, Japan) was used to capture the image of the FITC-labeled TUNEL-positive cells. A negative and a positive control were simultaneously prepared along with our treated experiments following the instructions. Moreover, the TUNEL assay was performed as a complementary method to flow cytometric assay in order to demonstrate the apoptosis level more accurately than flow cytometric assay alone. The cell nucleus was labeled in blue by DAPI (Invitrogen, Molecular Probes, Eugene, OR) and the nick-ends were labeled in green. A merge between the nucleus (blue) and nick-end (green) labeling showed up as purple. The positive section was pretreated with Dnase1 solution and the negative section with PBS before addition of the reaction mixture. And Figure S2 showed the negative and positive control of TUNEL assay.

### Statistical Analysis

All the data were expressed as mean±SD (standard deviation, SD) and all the statistical analysis was performed using SPSS statistical software package (SPSS Inc., Chicago, IL, USA). Furthermore, p<0.05 was considered statistically significant.

## Results

### Clinical characteristics

The clinical data were obtained from all the patients who participated in the study. Placenta tissues were classified into two groups: preeclampsia (PE, n=25) and normal pregnancy (N, n=25). Preeclampsia was diagnosed by the standard criteria while normal pregnancy was defined as not having preeclampsia or any other complications (including maternal history of hypertension and/or renal disease, maternal infection, smoking, alcholism, chemical dependency, and fetal congenital anomalies). 

### SPRY4-IT1 expression in preeclampsia and normal pregnant placenta tissues

**Figure 1:**
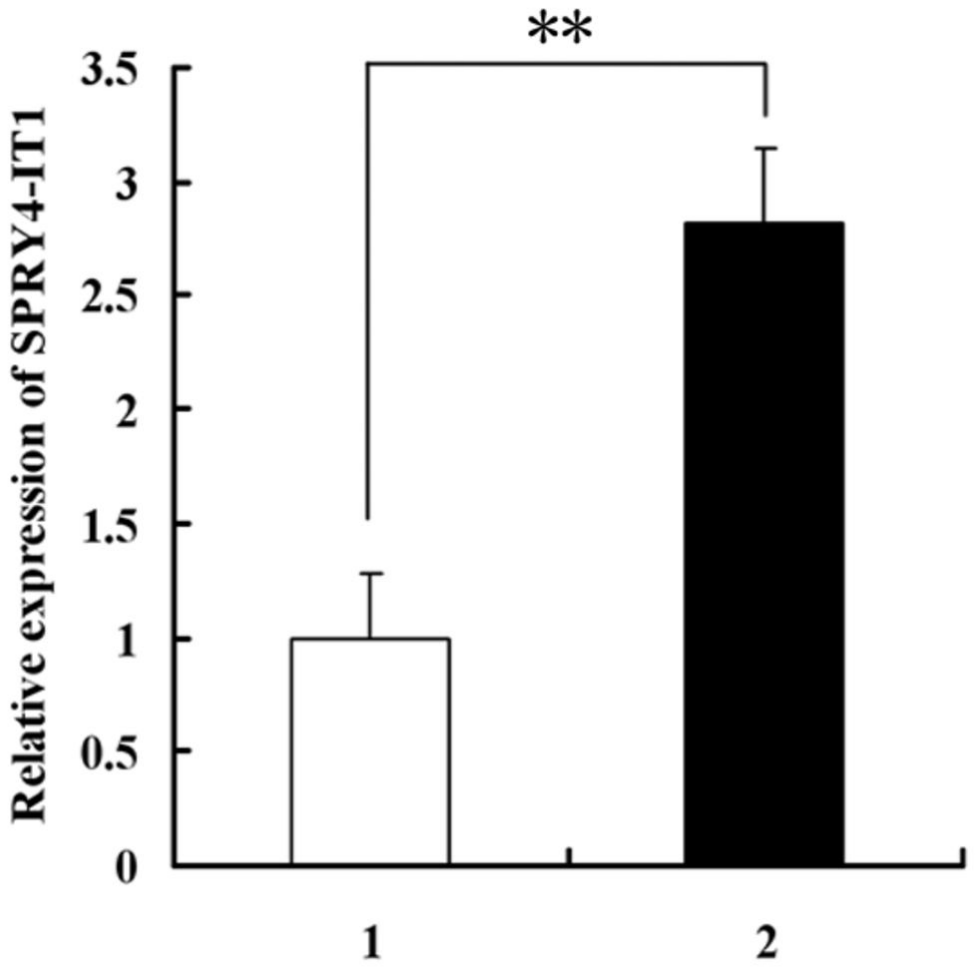
Relative expression of SPRY4-IT1 was higher in preeclampsia placenta tissues as compared to the normal pregnancies, as determined by qRT- PCR. Values are represented as mean±SEM (**: P<0.01).

A qRT-PCR analysis was conducted by comparing 25 preeclampsic placentas with 25 normal ones. The expression level of SPRY4-IT1 was 28% higher in the preeclampsic placentas as compared to that of the normal ones (Figure 1). Table 1 shows the patients’ clinical characteristics in details.

**Table 1:** Clinical characteristics of normal and preeclamptic pregnancies.

Parameters	PE (n=25)	N(n=25)	P ^a^ value Normals vs PE
Maternal age	30.5 ± 5.7	30.5±3.5	>0.05
Proteinuria (g/day)	>0.3	<0.3	<0.01
Gestational age (week)	35.2±3.7	39.0±1.2	>0.05
Systolic blood pressure, mm Hg	167 ± 20.1	114± 6.8	<0.01
Diastolic blood pressure, mm Hg	112 ± 12.8	73 ± 7.1	<0.01
Body weight of infant (g)	2552± 740	3392± 413	<0.05

All results are presented as mean ±SD (SD: standard deviation).

^a^ Obtained by 1-way analysis of variance using SPSS 18.0 software (SPSS Inc, Chicago, Illinois, United States of America)

### Overexpression and downregulation of SPRY4-IT1 in human trophoblast cells (HTR-8/SVneo)

**Figure 2:**
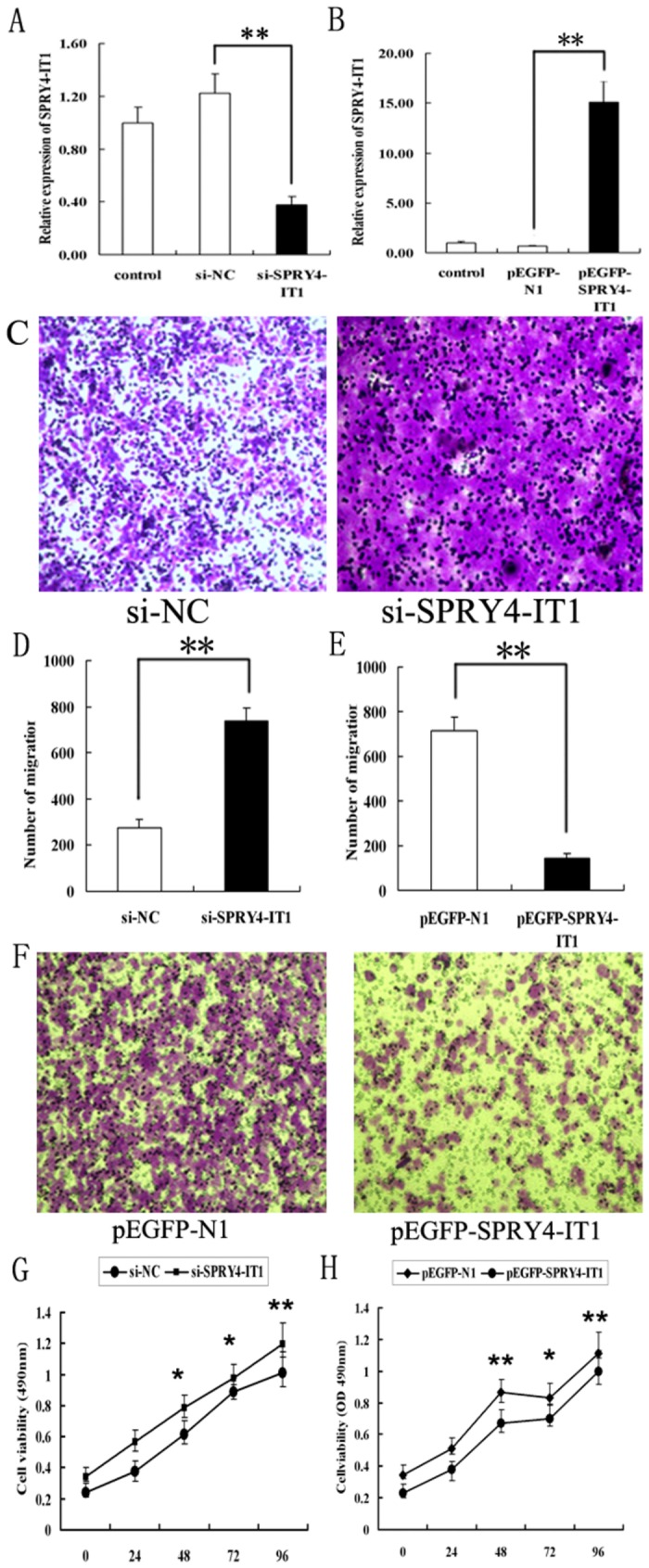
The effects of SPRY4-IT1 on HTR-8/SVneo cell proliferation and migration. The expression of SPRY4-IT1 in the HTR-8/SVneo cells transfected with siRNA targeting SPRY4-IT1 (A left) and plasmid overexpressing SPRY4-IT1 (A right), detected by qRT- PCR. B: The proliferation was increased in si-SPRY4-IT1 group as compared to that of the negative control (left) while it was decreased in the group with SPRY4-IT1 overexpression (right) as compared to the cells transfected with GFP as identified by MTT assays. C&D: The migration capacity of the cells transfected with si-SPRY4-IT1 was significantly higher than that of the negative control (C) and lower in the cells overexpressing SPRY4-IT1 (D), as determined by Transwell assays (Values are mean±SEM;*: P<0.05; **: P<0.01).

The HTR-8/SVneo cells that were sowed into the 6-well plates were transfected with si-RNAs or plasmids targeting SPRY4-IT1. They were then harvested after 48 hours for RNA extraction. The inhibition efficiency was assessed by qPCR, which was at 70% (Figure 2A, left, p<0.01) as compared to that of the negative control. However, they had a 15-fold upregulation by p-EGFP-SPRY4-IT1 (Figure 2A, right, p<0.01) as compared to that of the pEGFP-N1, respectively. Also, the expression level of GFP protein was tested to ensure a similar quantity of GFP in cells transfected with pEGFP-N1 and p-EGFP-SPRY4-IT1 by Western blotting, since the pEGFP-N1 vector over-expresses a fusion protein GFP (showed in Figure S1, p=0.2).

### Modulation of SPRY4-IT1 expression in cell migration and proliferation

It was determined that the migratory capacity of HTR-8/SVneo cells transfected with siRNAs targeting SPRY4-IT1 was significantly higher (2.6 fold, p<0.01) than those transfected with si-NC (Figure 2C&D, p<0.01). Meanwhile, the number of migrated cells overexpressing SPRY4-IT1 was specifically reduced by approximately 80% as compared to that of the negative control (Figure 2E&F, p<0.01). Also, the proliferation assays showed the same tendency (Figure 2G&H, p<0.05) with migration analysis. Therefore, these data proved that SPRY4-IT1 might be closely associated with the inhibition of migration behavior in HTR-8/SVneo cells. In addition, the cell invasion assay was performed but without positive results (no statistical difference between groups). 

### Effects of SPRY4-IT1 Expression on Cell Apoptosis

**Figure 3:**
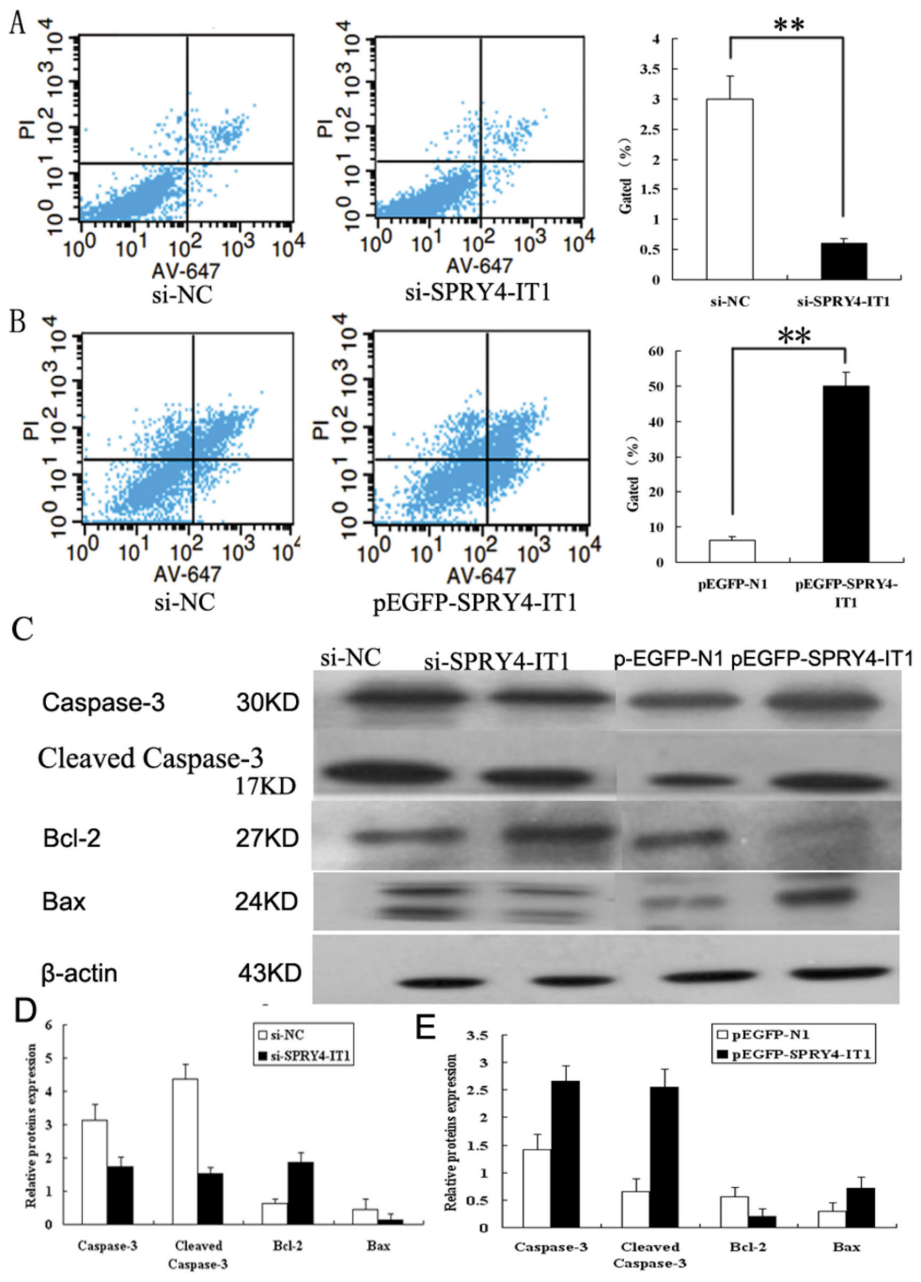
Cell apoptosis assays by flow cytometry and western blotting. A: Cells transfected with siRNA that targeted SPRY4-IT1 showed a significantly lower rate of apoptosis by flow cytometry. B: Cells transfected with plasmid overexpressing SPRY4-IT1 showed a significant increase in apoptotic rate as compared to that of the GFP as demonstrated by flow cytometry. C&D: Western blotting analysis of apoptotic protein in cells transfected with si-SPRY4-IT1 displayed a decrease of Caspase-3 (30KD), cleaved Caspase-3 (17KD) and Bax (24KD) while an increase of Bcl-2 (26KD) and cells overexpressing SPRY4-IT1 showed an increase in the expression of Caspase-3 and Bax and a reduction in the expression of Bcl-2 (Values are mean±SEM;*: P < 0.05; **: P < 0.01).

**Figure 4:**
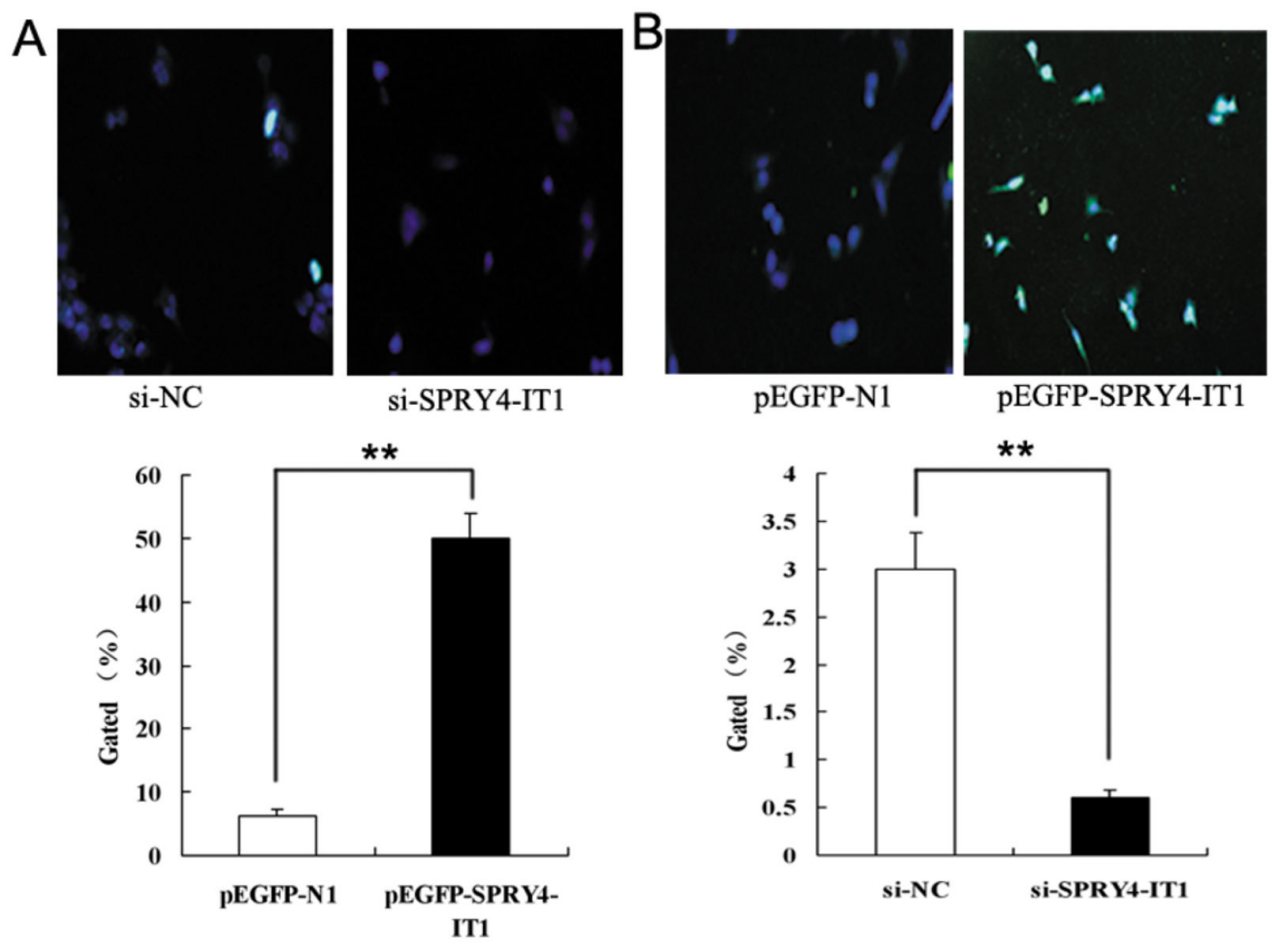
Cell apoptosis assays by TUNEL. A:.Cells treated with siRNA that targeted SPRY4-IT1 showed a significantly lower rate of apoptosis by TUNEL. B: Cells treated with plasmid overexpressing SPRY4-IT1 showed a significant increase in apoptotic rate as compared to that of the GFP as demonstrated by TUNEL assay (Values are mean±SEM;*: P < 0.05; **: P < 0.01).

Flow cytometry, TUNEL (terminal dexynucleotidyl transferase (TdT)-mediated dUTP nick end labeling), and western blotting analysis were used in order to obtain more accurate and comprehensive information about the impact of SPRY4-IT1 on cell apoptosis. When HTR-8/SVneo cells were transfected with si-RNAs targeting SPRY4-IT1, a significant decrease of apoptosis was observed as compared to those infected with si-NC using both flow cytometry (Figure 3A, p<0.01) and TUNEL (Figure 4B, p<0.01) assays. In contrast, the cells infected with p-EGFP-SPRY4-IT1 showed an apparent increase of apoptosis in contrast to those transfected with GFP (Figures 3B & 4C, p<0.01). On the other hand, Western blot analysis indicated that the expression level of Caspase-3 and Bax protein decreased in the cells treated with si-SPRY4-IT1 while they were increased with overexpression of SPRY4-IT1 (Figure 3C, p<0.01). The expression level of Bcl-2 protein presented reverse results (Figure 3C, p<0.01). 

### The role of SPRY4-IT1on network formation in vitro

**Figure 5:**
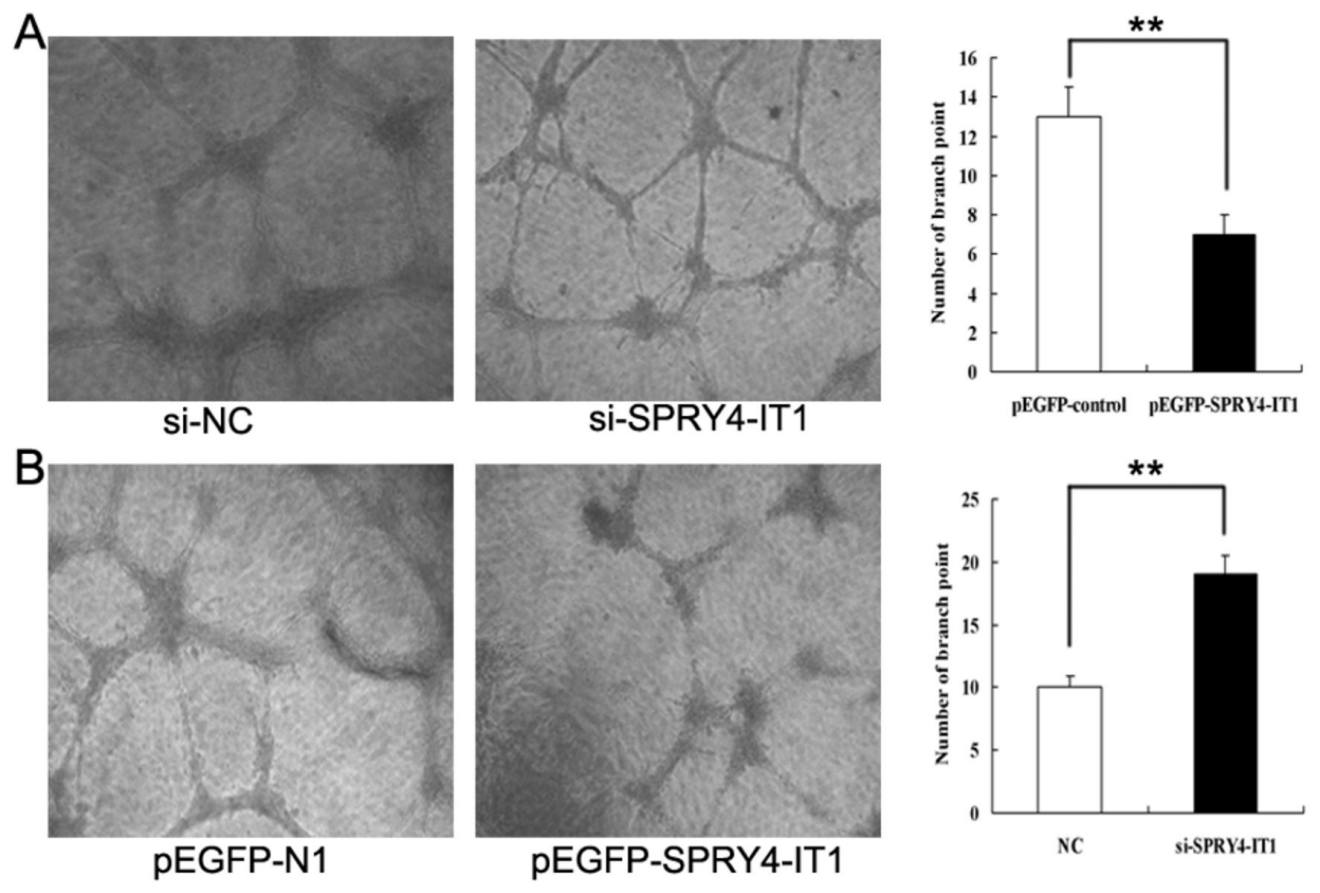
The effects of SPRY4-IT1 on HTR-8/SVneo cell network formation. A&B: Cells transfected with siRNA targeting SPRY4-IT1 showed an increase in node numbers as compared to the negative control while cells treated with plasmid overexpressing SPRY4-IT1 presented a decrease in node numbers as compared to that of the GFP (Values are mean±SEM; *: P < 0.05; **: P<0.01).

Compared with cells treated with overexpression plasmids of SPRY4-IT1, the network formation were significantly better in the cells treated with si-SPRY4-IT1, as calculated by counting the node numbers (Figure 5A&B, p<0.01). 

## Discussion

It has been demonstrated by cDNA cloning studies [28] and genomic microarray analysis [29] that more than 90% of the human genome transcriptional products do not code for proteins [30], which are referred to as non-protein coding RNAs (ncRNAs). Among these, microRNAs (miRNA) have been certified as important biological RNAs in the post-transcriptional regulation of the target genes. Besides, lncRNAs (200~100000 nt) as a newly discovered class of noncoding genes, are another crucial member of these biological RNAs. Although the functional significance of miRNAs has been adequately evidenced on inhibiting target gene expression through posttranscriptional regulation, little is known about the functionality of most lncRNAs. As a novel molecule in the ncRNA world, lncRNA has become well-known by emerging evidence for its contribution to embryonic development and tumorigenesis. Remarkable progress of lncRNAs has indicated the regulatory and structural roles in important biological processes, such as X-chromosome inactivation [31,32], genomic imprinting [33], cell differentiation and proliferation, modulation of migration [34],and apoptosis [35,36], through diverse molecular mechanisms [37,38]. However, there is little information concerning the biological and molecular mechanisms of lncRNAs underlying the pathogenesis of preeclampsia. 

In this study, we found a higher expression of SPRY4-IT1 in 25 human preeclampsic placental tissues as compared with the normal pregnancies. SPRY4-IT1 (708 bp), which was localized in 5q31.3, was derived from an intronic region within the SPRY4 gene and was predicted to contain several long hairpins in its secondary structure, which was originally reported by Divya Khaitan and colleagues to play an important role in melanoma pathogenesis in humans [19]. In their study (Divya Khaitan), the knockdown of SPRY4-IT1 expression resulted in cell growth defects, decreased invasion and migration, and increased rates of apoptosis in melanoma cells whereas converse results were observed for a melanoma cell line overexpressing SPRY4-IT1. In this study, we focused on the identification and characteristics of lncRNAs SPRY4-IT1 in preeclampsia and its role in migration, invasion, and apoptosis rate of trophoblast cells HTR-8/SVneo. However, the in vitro data in our study proved that the migration rate and proliferation of the HTR-8/SVneo cells were increased and their apoptosis rate was decreased when lncRNA SPRY4-IT1 was inhibited. However, reverse results were observed in HTR-8/SVneo cells when SPRY4-IT1 was overexpressed. Taken together, we suggested that SPRY4-IT1 might play an important part in proliferation, migration, and apoptosis of trophoblast cells in preeclampsia. However, this phenomenon was not consistent with the article reported by Khaitan conducted in melanoma. In their study [19], they found a decreased cell migration and an increased rate of apoptosis for the SPRY4-IT1, knocked-down in melanoma cells. However, they reported a converse increase in the speed of migration for plasmids overexpressing the SPRY4-IT1. The LncRNAs appeared significant tissue and cell specificity [39], so they might show various phenotypes in different tissues and different stages of life process. Thus, trophoblast and cancer cells probably present different biological behavior in preeclampsia and in tumor, respectively. For example, elevation of HOTAIR promotes invasiveness and metastasis in the primary breast tumor [22]; however, it decreases cell proliferation and induces apoptosis [21] in the pancreatic cancer cells.

Generally, the disorder of maternal spiral arteries remodeling might contribute to the pathogenesis of preeclampsia. For network formation assays, the network in cells transfected with si-SPRY4-IT1 was better than that of the negative control, while the cells overexpressing SPRY4-IT1 showed a worse network as compared to that of the control. Together these findings indicated that endometrial epithelial cells might act upon the trophoblast cells to promote the requisite cellular steps for the trophoblast conversion of maternal spiral arteries. Therefore, the disrupted expression of SPRY4-IT1 might cause implications for inadequate conversion of maternal spiral arteries, leading to placental abnormalities or preeclampsia. Thus, we postulated that the lncRNA SPRY4-IT1 might also play a role in the process of spiral artery remodeling in preeclampsia. In addition, although we tested the expression level of several angiogenic factors, such as vascular endothelial growth factor (VEGF), angioprotein 1 (Ang1), and angioprotein 2 (Ang2), no change was found in the cells after being transfected with siRNAs or plasmids targeting SPRY4-IT1 (data not shown). So the detailed mechanism of SPRY4-IT1 involved in the remodeling of the spiral arteries in placenta in vivo requires to be further clarified in details. 

Although the lncRNA SPRY4-IT1 might have biological impact on the behavior of the preeclamptic trophoblast cells, as known that one lncRNA could regulate multiple functions of cells, whereas one cell function may also be controlled by multiple lncRNAs. Thus, other target lncRNAs maybe involved in the modulation of trophoblast cells’ functions and further studies will be needed to clarify this point. In addition, the basis for the molecular mechanisms of lncRNAs is still being elucidated [40]. The study of the molecular mechanisms of lncRNAs is really time consuming and there have been only a few diverse hypothetical mechanisms [41,42] proposed by which the lncRNAs could exert their effects: (1) lncRNAs guiding the chromatin-modifying complexes such as PRC2, LSD1, and DNA methyltransferases to specific genomic loci in cis and in trans[43,44]; (2) The binding of lncRNAs to miRNAs and preventing specific miRNAs from binding to their target mRNAs [45]; (3) lncRNAs regulating transcriptional factors by directly binding to their promoters [46,47]. However, which of the above could present the mechanisms of lncRNAs SPRY4-IT1 in the regulation of the preeclamptic trophoblast cells remain to be further elucidated.

In conclusion, we identified that besides lncRNA H19 [48], SPRY4-IT1 could also participate in the pathogenesis of preeclampsia. However, the exact upstream regulatory mechanisms of SPRY4-IT1 remain to be elucidated in order to provide a new target for and early diagnosis and treatment of preeclampsia.

## Supporting Information

Figure S1 **The expression of GFP protein showed no difference between cells transfected with pEGFP-N1 and pEGFP-SPRY4-IT1.** (Values are mean±SEM, p=0.2).
(TIF)

Figure S2 **The negative and positive control of TUNEL assay.**
(TIF)
