# Supplementary figures and images for: Upregulation of Long Noncoding RNA SPRY4-IT1 Modulates Proliferation, Migration, Apoptosis, and Network Formation in Trophoblast Cells HTR-8SV/neo
Source: PLoS One. 2013 Nov 6;8(11):e79598. doi: 10.1371/journal.pone.0079598 (PMC3819274; doi:10.1371/journal.pone.0079598)

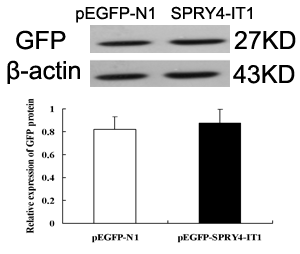

Supplement: Figure S1 — The expression of GFP protein showed no difference between cells transfected with pEGFP-N1 and pEGFP-SPRY4-IT1. (Values are mean±SEM, p=0.2). (TIF) [file pone.0079598.s001.tif]

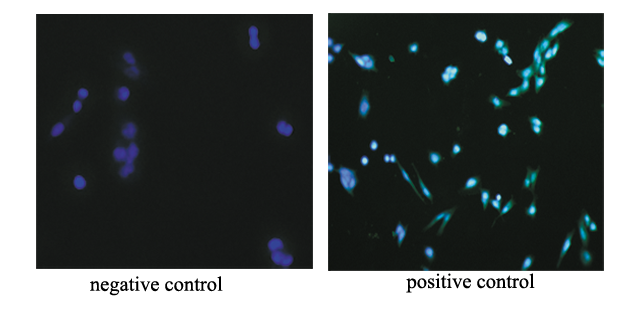

Supplement: Figure S2 — The negative and positive control of TUNEL assay. (TIF) [file pone.0079598.s002.tif]
